# Healthcare Disparities and Outcomes of Cancer Patients in a Community Setting from a COVID-19 Epicenter

**DOI:** 10.3390/curroncol29020098

**Published:** 2022-02-16

**Authors:** Brianna M. Jones, Eric J. Lehrer, Anurag Saraf, Zahra Shafaee, Lucas Resende Salgado, Virginia W. Osborn

**Affiliations:** 1 Department of Radiation Oncology, Icahn School of Medicine at Mount Sinai, New York, NY 10029, USA; eric.lehrer@mountsinai.org; 2 Harvard Radiation Oncology Program, Massachusetts General Brigham, Boston, MA 02115, USA; asaraf1@mgh.harvard.edu; 3 Department of Radiation Oncology, Massachusetts General Hospital, Boston, MA 02114, USA; vwosborn@mgh.harvard.edu; 4 Department of Surgery, Elmhurst Hospital Center, New York, NY 11373, USA; shafaeez1@nychhc.org; 5 Department of Radiation Oncology, Elmhurst Hospital Center, New York, NY 11373, USA; resendel2@nychhc.org

**Keywords:** COVID-19, cancer, outcomes, health disparities, mortality, severity

## Abstract

There have been numerous studies demonstrating how cancer patients are at an increased risk of mortality. Within New York City, our community hospital emerged as an epicenter of the first wave of the pandemic in the spring of 2020 and serves a unique population that is predominately uninsured, of a lower income, and racially/ethnically diverse. In this single institution retrospective study, the authors seek to investigate COVID-19 diagnosis, severity and mortality in patients with an active cancer diagnosis. Demographic, clinical characteristics, treatment, SARS-CoV-2 laboratory results, and outcomes were evaluated. In our community hospital during the first wave of the COVID-19 pandemic in the United States, patients with active cancer diagnosis appear to be at increased risk for mortality (30%) and severe events (50%) due to the SARS-CoV-2 infection compared to the general population. A higher proportion of active cancer patients with Medicaid insurance, Hispanic ethnicity, other race, and male sex had complications and death from COVID-19 infection. The pandemic has highlighted the health inequities that exist in vulnerable patient populations and underserved communities such as ours.

## 1. Introduction

The novel coronavirus disease 2019 (COVID-19) pandemic due to SARS-CoV-2 virus has resulted in over 5 million deaths worldwide with over 4 million cases diagnosed in New York State alone [1]. New York City (NYC) had its first confirmed case diagnosed on 29 February 2020 and in the subsequent months the city’s hospitals were inundated with infected patients [2]. Within New York City, Elmhurst Hospital Center (EHC) emerged as an epicenter [3]. At the peak on 23 March 2020 there was an 83.1% test positivity rate at EHC [4]. EHC is a 545 bed public hospital and serves one of most racially and ethnically diverse populations in the United States with a catchment zone of roughly 1.2 million residents of Queens [5]. The patients seen at EHC are predominantly uninsured or with medical coverage provided to people with a limited income or people over the age of 65 or with disabilities, such as Medicaid or Medicare, respectively. Several studies have noted how socioeconomic and racial disparities contribute to poorer outcomes [6,7,8,9]. Given the disproportionate burden of morbidity and mortality on communities with a significant proportion of racial and ethnic minority groups, studies that adequately represent these high risk populations are paramount.

In NYC, there was an enormous shift in delivery of cancer care during the height of the COVID outbreak. Non-emergent surgeries and some diagnostic procedures were postponed or redirected elsewhere, as operating rooms were converted to accommodate acutely ill patients. Radiation was transitioned to more short course/hypofractionated regimens and certain populations were encouraged to delay or even completely omit treatment [10,11]. On the other hand, radiation was also utilized for some patients that otherwise would have been managed surgically due to surgical staff reassignment and concern for intraoperative risk [12]. Chemotherapy was switched from intravenous infusions to oral chemotherapy regimens and immunotherapy was altered on a case-by-case basis [13,14]. Any indirect outcomes of these modifications will need to be evaluated in future studies. While the data are still evolving, cancer patients appear to be a vulnerable population to the SARS-CoV-2 virus [15,16,17,18,19,20]. There have not been many studies that have evaluated outcomes in patients with COVID and an active cancer diagnosis or recent treatment.

In the current study, the authors seek to determine COVID-related outcomes in patients with an active cancer diagnosis at Elmhurst Hospital Center (EHC) in the first wave of the pandemic. There have been studies evaluating outcomes in cancer patients in academic centers [2,21,22,23,24], but the high community infection rates and diverse population in our public health system in NYC provide a unique window into the effects of the disease within one of the hardest-hit communities to date.

## 2. Materials and Methods

### 2.1. Inclusion

A retrospective analysis of patients at Elmhurst Hospital Center with active cancer between January 2020 and June 2020 was performed via chart review (BMJ, VWO, ZS). Inclusion criteria included: diagnosis via pathology report and/or receipt of oncologic treatment (including surgery, radiation therapy or systemic therapy) during the period of the COVID pandemic at Elmhurst Hospital, defined for these purposes as 1 January–30 June 2020. Patients with benign tumors were excluded. We obtained data from the electronic medical record (EMR) at EHC on demographic, clinical characteristics, cancer diagnosis, staging, pathology, treatment, COVID laboratory results, and outcomes. Patients without a COVID laboratory results available in the EMR were subsequently excluded from statistical analysis. This study was reviewed and approved by our institutional review board (IRB) review and informed consent was waived.

### 2.2. Definitions

Active cancer was defined as those with recent diagnosis of cancer (via confirmed pathology) or those undergoing active cancer-directed therapy, including radiation, surgery or chemotherapy within the data collection period (benign tumors excluded). COVID diagnosis was determined by laboratory evidence of SARS-CoV-2 using RT-PCR testing on nasopharyngeal or oropharyngeal swabs. COVID-confirmed mortality was defined as death within 30 days of confirmed laboratory evidence for SARS-CoV-2 using RT-PCR testing on nasopharyngeal swabs. Severe events were defined as patients with laboratory-confirmed SARS-CoV-2 requiring hospitalization, oxygen supplementation, mechanical ventilation, or death within 30 days of diagnosis.

### 2.3. Statistics

Statistical analysis was performed using an R Studio. Demographics, clinical presentation, and outcomes were analyzed using descriptive statistics, Chi square, Fischer’s exact and standard t-tests. The prevalence of COVID diagnosis and rates of severe events and death were the primary outcomes of this study. Predictors of incidence and prognostic factors for COVID mortality and severity were determined using univariate logistic regression.

Univariate logistic regression models were constructed, where the dependent variable was a binary outcome. Odds ratios and their associated 95% confidence intervals were constructed, where the null hypothesis was rejected for *p* < 0.05. Statistical analyses were conducted using R Studio Version 1.1.383; the “aod” and “ggplot2” packages were utilized. Multivariate analyses were not performed due to the low incidence of COVID-related severe events and mortality.

## 3. Results

### 3.1. Patients

Two hundred sixty-six patients were identified with active cancer at Elmhurst Hospital Center from January 2020 to June 2020. Of those 266 patients, 111 had a SARS-CoV-2 PCR test reported in EHR. The remaining 155 patients with active cancer diagnosis and no available SARS-CoV-2 PCR test were excluded from further analysis. Of the 266 patients, 20 (7.5%) had laboratory-confirmed COVID diagnosis, 10 (3.8%) had severe COVID, and 6 (2.3%) died from COVID. The most common presenting symptoms: fever (60%, 12), cough (45%, 9), dyspnea (40%, 8), nausea/vomiting (10%, 2), fatigue or malaise (5%, 1), diarrhea (5%, 1) asymptomatic (15%, 3). See Table 1 for further demographic and clinical characteristics of the 111 patients with an active cancer diagnosis and COVID-19 test.

Median follow-up for the entire cohort from time of diagnosis to time of data collection cutoff was 28 weeks. The median age of the COVID-negative cohort was 60 (IQR 52–69) and COVID-positive patients were 61 years old (IQR 52–68). Males represented 46.2% of the non-COVID cohort. There more male patients in the COVID-positive cohort (65%), severe COVID (70%), and COVID-confirmed deaths (83.3%).

In the COVID negative cohort, 42.9% were of Hispanic ethnicity with racial breakdown as follows: 4.4% White, 13.2% Black, 23.1% Asian, 1.1% American Indian/Alaskan native, 53.8% other, 4.4% unknown. A higher proportion of COVID-positive patients (65%), severe COVID (60%), and COVID-confirmed deaths (83.3%) were of Hispanic ethnicity compared to cancer patients without COVID (42.9%). 65% of patients with COVID had Medicaid, 80% of patients with severe COVID, 83.3% of patients who died from COVID as compared to 65.9% of patients in the non-COVID cohort. 100% of patients who died of COVID had either Medicaid or Medicare.

In the overall cohort who took a COVID test, 63 patients had surgery during this time period, 18 received radiation therapy, and 63 received cytotoxic chemotherapy. Intent of therapy was curative in 76 patients and palliative in 35 patients; 13 patients had a cancer diagnosis but had not received any cancer directed therapy at time of data collection. In the entire cohort of 266 active cancer patients, 177 patients did not have any delay, interruption or change in treatment, while 89 (33.5%) patients did experience a treatment modification. Sixty-one treatments were delayed, 38 had treatment interruption, 16 had treatment change. Four patients were transferred to a different facility for a portion of cancer care.

A majority of the COVID-positive patients had solid (80%) versus hematologic (20%) malignancies. Five out of sixteen (31%) patients with solid cancer died of COVID and one out of four (25%) patients with hematologic cancer died. There were 3 patients who had surgery, 5 who received radiation, and 15 received cytotoxic chemotherapy. Of the 6 COVID-confirmed deaths, 3 received radiation (50%), and 5 (66.7%) received cytotoxic chemotherapy. Refer to Table 1 for demographic and clinical factors. There were no significant associations between demographic and clinical factors with COVID diagnosis, severity, or mortality. Although, there was a trend towards statistical significance for patients with hypertension and COVID diagnosis (*p* = 0.05, see Table 2). Intent of therapy was curative in majority of COVID-positive (85%), severe-COVID (80%), and COVID-confirmed deaths (83%).

### 3.2. Treatment and Mortality

There were 10 patients hospitalized for COVID with 1 patient requiring intensive care unit admission. Of the 20 patients with confirmed COVID, 6 were treated with medication (i.e., hydroxychloroquine, azithromycin), 12 with supportive care alone, and 8 required supplemental oxygen. The 30-day overall mortality rate for non-COVID patients was 6.6% and 30% for COVID patients. In the entire initial cohort (*n* = 266), there were 6 confirmed COVID-related deaths (2.3%). Of those who tested positive, 10 had severe events (50%) and 6 had confirmed COVID-related deaths (30%). There were no significant predictors of COVID diagnosis, severity, or mortality (see Table 3).

## 4. Discussion

Our single institutional experience shows the high case fatality rates in patients with active cancer treatment and COVID-19 diagnosis. Among the cancer patients with laboratory-confirmed COVID, a third of patients died and half had severe events requiring admission, supplemental oxygen, and/or mechanical ventilation. This study contributes to the evolving literature on the impact of COVID in patients with an active cancer diagnosis. The strengths of this study include strict inclusion criteria of an active cancer diagnosis, a non-COVID comparison group, and a community setting. Moreover, this study provides COVID outcomes in the context of an urban, diverse, public health system in an epicenter of the first wave of the COVID-19 pandemic in the United States.

Interestingly, other tertiary care centers in NYC reported a lower case fatality rate (CFR) of 11–12% [2,21,22,23,24]. The average COVID mortality for all cancer patients based on available data in Table 4 is 19.4% and 22.6% when including studies with hematologic cancers. The exact reasons for these differences in CFR are unclear, but it could reflect health care inequities that exist in NYC and throughout the world. Institutions with a lower CFR were more likely to be private academic centers that serve generally different patient populations than the New York Health and Hospital health system. Poor surge capacity and lack of resources (e.g., personal protective equipment, ventilators, nonrebreathers etc.) at EHC, particularly in March and April, may explain our high case-fatality rate. Notably, all the COVID-related deaths in our cohort occurred between 20 March–28 April 2020. It is possible that mortality rates have improved over time for subsequent waves of the pandemic due to inadequate resources, public awareness of COVID-19, widespread vaccination, and improved medical management of COVID-19. In a study by Chavez-MacGregor et al. [25], mortality and hospitalization rates of cancer patients with COVID decreased towards the end of 2020. A recent European registry analysis (On COVID) of 2634 patients with cancer diagnosed with COVID, also showed that mortality has improved in subsequent waves of the pandemic, which is reassuring and temporal trends in COVID outcomes should continue to be evaluated [26].

The disproportionate burden of morbidity and mortality from COVID in racial and ethnic minorities has been well-documented and should elicit further investigation [6,7,8,9,27,29,30,43,48]. According to the New York State Department of Health, approximately 34% and 28% of COVID fatalities are Hispanic or Black, despite comprising 29% and 22% of the population, respectively [49]. This is compounded with the known propensity to worse cancer outcomes based on race and insurance status, as well as underrepresentation of minorities in cancer clinical trials [50,51,52]. In this study, it is notable that 60% of severe COVID-related events and 83% of COVID deaths in our cohort were of Hispanic ethnicity, despite representing 54% of our entire population of active cancer patients and 42.6% of active cancer patients with a COVID laboratory test. Additionally, 88% of severe events and 83% of deaths had Medicaid insurance compared to 65% of the COVID-negative cohort. We did not find a statistically significant association with race, ethnicity, or insurance status with COVID incidence, severity, or mortality, which could be related to the small sample size.

In view of the high COVID-related mortality and severity rates in our cohort of patients with active cancer during the first wave of the pandemic in NYC, a recent cancer diagnosis or active cancer treatment is likely associated with worse outcomes. A majority of previous studies included patients with current or historical diagnosis of cancer, whereas our study and a few others have focused on patients with an active cancer diagnosis [20,25,27]. In one of the largest retrospective case-control analyses to date, Wang and colleagues [27] found an increased risk of COVID-19 infection, hospitalization, and death in patients with recent cancer diagnosis as compared to patients without cancer. Another cohort study found patients with recent cancer treatment (within 3 months of COVID diagnosis) and COVID had significantly higher risk of adverse outcomes than patients with no recent cancer treatment and in patients without cancer [25]. In fact, cancer patients without recent treatment had outcomes similar to patients without cancer [25]. Several other studies have previously noted receipt of anticancer therapy within 4 weeks of diagnosis of COVID was associated with adverse outcomes [20,30,37,53,54]. It is notable that patients who died from COVID in our cohort received anticancer therapy with the median time interval of 2 weeks of diagnosis (See Supplementary Table S1 for more details). There were no significant predictors for COVID diagnosis, severity, or mortality in our cohort of active cancer patients. However, there was a trend towards significance for hypertension (*p* = 0.05), which has been previously demonstrated in larger case series [20,39,55]. It is clear that patients with cancer represent a heterogeneous group and particular attention to COVID patients with recent diagnosis and treatment is warranted.

There are obvious limitations to this study as a small, single institution cohort, but this is important nonetheless given the continued outbreaks of COVID throughout the world and because of the relative lack of data that exists in regards to COVID outcomes in patients with active cancer diagnosis in racially and ethnically diverse patient populations. The high mortality rate among patients with confirmed COVID diagnosis may be in part due to limited testing of patients; only 42% of our initial cohort had a COVID test. Moreover, during the early portion of the time studying SARS-CoV-2 RT-PCR, testing was reserved for patients with moderate to severe symptoms, which may contribute to the elevated case fatality rate in our population. Therefore, the elevated mortality rate found in this study may be in part due to preferential testing in patients sufficiently ill to require SARS-CoV-2 testing. It is also possible that data collection for testing and hospitalization did not capture all patients, as many were redirected to other hospitals during the height of the pandemic or were tested in outside clinics. Long-term oncologic outcomes will also be important to evaluate in this population with comparison to patients from previous years. Future studies should be dedicated to evaluating both COVID-related and oncologic long-term outcomes in these vulnerable patients, especially in the context of profound changes in cancer care delivery during the pandemic [56,57].

EHC serves an incredibly diverse and predominantly poor, uninsured, underserved population that faces a multitude of economic, cultural, and language barriers. In diverse and historically underserved communities such as Queens, the first wave of the pandemic exposed the vulnerability of these communities and the healthcare inequities that persist in the United States. In view of new highly infectious variants associated with lower vaccine effectiveness, such as B.1.617.2 (delta) and B.1.1.529 (omicron), COVID continues to pose a significant threat to vulnerable populations [58,59]. Resource allocation for public and safety-net hospitals and public health policies to mitigate disparate COVID-related outcomes in underserved communities is necessary.

## 5. Conclusions

In this retrospective cohort study of a diverse community hospital during the first wave of the pandemic, we found that patients with an active cancer diagnosis and COVID had high rates of severe events (50%) and mortality (30%). A disproportionate number of active cancer patients with Medicaid insurance, Hispanic ethnicity, other race, and male sex had adverse COVID-related complications or death. There were no significant predictors of COVID diagnosis, severity, or mortality.

## Figures and Tables

**Table 1:** Demographics and clinical characteristics in entire cohort.

	COVID Negative	COVID Positive	COVID Severe Events	COVID Deaths
Patients	91 (82.0%)	20 (18.0%)	10 (9.0%)	6 (5.4%)
Median Age (*n*, range)	60 (29–85)	61 (30–80)	62 (40–80)	61 (40–80)
Gender				
Male	42 (46.2%)	13 (65.0%)	7 (70.0%)	5 (83.3%)
Female	49 (53.8%)	7 (35.0%)	3 (30.0%)	1 (16.7%)
Hispanic/Ethnicity	39 (42.9%)	13 (65.0%)	6 (60.0%)	5 (83.3%)
Race				
Asian	21 (23.1%)	3 (15.0%)	2 (20.0%)	1 (16.7%)
African-American	12 (13.2%)	2 (10.0%)	1 (10.0%)	0
White	4 (4.4%)	0	0	0
Other	49 (53.8%)	14 (70.0%)	7 (70.0%)	5 (83.3%)
American Indian/Alaska native	1 (1.1%)	0	0	0
Unknown	4 (4.4%)	1 (5.0%)	0	0
Insurance				
Medicaid	60 (65.9%)	13 (65.0%)	8 (80.0%)	5 (83.3%)
Medicare	6 (6.6%)	2 (10.0%)	1 (10.0%)	1 (16.7%)
Commercial	24 (26.4%)	4 (20.0%)	1 (10.0%)	0
None	0	1 (5.0%)	0	0
Other *	1 (1.1%)	0	0	0
Current/Former Smokers	29 (31.9%)	6 (30.0%)	5 (50.0%)	3 (50.0%)
Any comorbidity	56 (61.5%)	11 (55.0%)	6 (60.0%)	3 (50.0%)
Solid malignancy	83 (91.2%)	16 (80.0%)	8 (80.0%)	5 (83.3%)
Hematologic malignancy	8 (8.8%)	4 (20.0%)	2 (20.0%)	1 (16.7%)
Curative intent	59 (64.8%)	17 (85.0%)	9 (90.0%)	5 (83.3%)
Cytotoxic Chemotherapy	49 (53.8%)	14 (70.0%)	7 (70.0%)	4 (66.7%)
Immunotherapy	10 (11.0%)	1 (5.0%)	0	0
Targeted therapy	14 (15.4%)	4 (20.0%)	2 (20.0%)	0
Radiation	13 (14.3%)	5 (25.0%)	4 (40.0%)	3 (50.0%)
Surgery or Biopsy	60 (65.9%)	3 (15.0%)	2 (20.0%)	1 (16.7%)
Treatment change	39 (42.9%)	16 (95.2%)	9 (87.5%)	5 (83.3%)
Mortality	6 (6.6%)	6 (30.0%)	6 (75.0%)	
Severe events		10 (50.0%)		

* Military, union, prison, workers’ compensations.

**Table 2:** Associated demographic and clinical factors with COVID positivity, severity, and mortality.

	COVID Diagnosis (*n* = 20)	COVID Severity (*n* = 10)	COVID Mortality (*n* = 6)
	*p*-Values		
Age	0.40	0.45	0.87
Male gender	0.15	0.19	0.10
Ethnicity (Hispanic vs. Non-Hispanic)	0.62	1.00	0.40
Other	0.22	0.75	0.22
Asian	0.56	1.00	1.00
African-American	1.00	1.00	1.00
White	1.00	1.00	1.00
Unknown	1.00	1.00	1.00
American Indian/Alaskan native	1.00	1.00	1.00
Insurance type	1.00	0.49	0.66
Current/Former Smokers	0.61	0.14	0.33
Any comorbidity	0.62	1.00	0.68
Diabetes	0.60	0.30	0.39
Hypertension	0.05	0.51	0.10
Cardiovascular disease	0.30	1.00	1.00
COPD	1.00	1.00	1.00
Kidney disease	1.00	0.63	0.59
Solid malignancy	0.22	0.26	0.45
Curative intent	0.11	0.16	0.66
Performance status	0.46	0.34	1.00
Systemic therapy	0.31	0.74	1.00
Cytotoxic chemotherapy	0.22	0.51	0.69
Radiation	0.40	0.11	0.09
Surgery or Biopsy	0.21	0.32	0.42

Fischer exact test used to determine ORs. *p*-value < 0.05 determined significant on two tailed test.

**Table 3:** Predictors of COVID diagnosis, severity, and mortality.

	Diagnosis		Severity		Mortality	
Variable	OR (95% CI)	*p*-Value	OR (95% CI)	*p*-Value	OR (95% CI)	*p*-Value
**Age**	0.99 (0.95–1.03)	0.69	1.05 (0.98–1.14)	0.18	1.02 (0.95–1.10)	0.66
**Sex**						
Male	Ref	Ref	Ref	Ref	Ref	Ref
Female	0.44 (0.16–1.23)	0.132	0.64 (0.09–4.10)	0.64	0.26 (0.01–2.28)	0.28
**Race**						
White/Caucasian	Ref	Ref	Ref	Ref	Ref	Ref
Black	Inf	1	Ref	Ref	Ref	Ref
Asian	Inf	1	2.00 (0.42–120.28)	0.71	Inf	1
Other	Inf	1	0.88 (0.03–24.99)	0.93	Inf	1
**Ethnicity**						
Non-Hispanic	Ref	Ref	Ref	Ref	Ref	Ref
Hispanic	1.42 (0.63–4.39)	0.50	0.64 (0.09–4.10)	0.64	3.75 (0.44–82.23)	0.28
**Insurance Type**						
Medicaid	Ref	Ref	Ref	Ref	Ref	Ref
Medicare	1.54 (0.11–0.38)	0.62	0.63 (0.02–18.29)	0.76	1.60 (0.05–47.12)	0.76
Commercial	0.77 (0.21–7.60)	0.67	0.21 (0.01–2.15)	0.22	Inf	1
None/Self Pay	Inf	1	Inf	1	Inf	1
Other	Inf	1	Ref	Ref	Ref	Ref
**Diabetes**						
No	Ref	Ref	Ref	Ref	Ref	Ref
Yes	1.43 (0.51–3.83)	0.49	2.33 (0.38–16.28)	0.37	0.56 (0.07–4.02)	0.55
**HTN**						
No	Ref	Ref	Ref	Ref	Ref	Ref
Yes	0.24 (0.01–1.31)	0.05	2.67 (0.38–24.34)	0.34	2.78 (0.32–61.34)	0.41
**CVD**						
No	Ref	Ref	Ref	Ref	Ref	Ref
Yes	0.24 (0.01–1.31)	0.18	Inf	1	Inf	1
**Immunocompromised**						
No	Ref	Ref	Ref	Ref	Ref	Ref
Yes	0.91 (0.05–6.05)	0.93	Inf	1	Inf	1
**COPD**						
No	Ref	Ref	Ref	Ref	Ref	Ref
Yes	Inf	1	Ref	Ref	Ref	Ref
**Asthma**						
No	Ref	Ref	Ref	Ref	Ref	Ref
Yes	Inf	1	Ref	Ref	Ref	Ref
**Comorbidity**						
No	Ref	Ref	Ref	Ref	Ref	Ref
Yes	0.76 (0.29–2.07)	0.59	1.5 (0.25–9.35)	0.65	0.75 (0.10–5.37)	0.77
**Solid vs. Hematologic Malignancy**						
Solid	Ref	Ref	Ref	Ref	Ref	Ref
Hematologic	2.59 (0.63–9.34)	0.16	1.00 (0.09–10.10)	1	0.73 (0.03–7.57)	0.81
**PS**						
KPS 70+ or ECOG 0 or 1	Ref	Ref	Ref	Ref	Ref	Ref
KPS < 70 or ECOG 2+	Inf	1	Inf	1	Inf	1
**Surgery**						
No	Ref	Ref	Ref	Ref	Ref	Ref
Yes	0.31 (0.07–1.01)	0.08	2.25 (0.18–54.04)	0.54	1.20 (0.05–15.65)	0.89
**Radiation**						
No	Ref	Ref	Ref	Ref	Ref	Ref
Yes	2.00 (0.57–6.22)	0.25	6.00 (0.68–133.97)	0.15	6.00 (0.70–65.95)	0.11
**Chemotherapy**						
No	Ref	Ref	Ref	Ref	Ref	Ref
Yes	2.57 (0.91–8.45)	0.09	0.58 (0.06–4.54)	0.61	0.54 (0.06–5.30)	0.58

**Table 4:** Summary of previously reported rates of COVID mortality and severity.

	Patients *	Number of Patients	Clinical Setting	Case Fatality Rate	Severe Event Rate **	Country
Current study	All active cancer patient	111	Inpatient and outpatient	30%	50%	US
Wang et al. [27]	All active cancer patients	73,449,510	Inpatient and outpatient	15%	48%	US
Chavez-MacGregor et al. [25]	All active cancer patients	507,307	Inpatient and outpatient	8%	34%	US
Sharafeldin et al. [28]	All cancer patients (N3C)	398,579	Inpatient and outpatient	2%	5.6%	US
Fillmore et al. [29]	All cancer patients	22,914	Inpatient and outpatient	11%	44%	US
Grivas et al. [30]	All cancer patients	4966	Inpatient and outpatient	14%	58%	US
Palmieri et al. [31]	All cancer patients	1797	Inpatient	35%		UK
Rüthrich et al. [32]	All cancer patients	435	Inpatient and outpatient	23%	55%	
Lee et al. [20]	All active cancer patients (UKCCMP)	800	Inpatient and outpatient	28%	45%	UK
Dai et al. [19]	All cancer patients	105	Inpatient and outpatient	11%		China
Robilotti et al. [21]	All cancer patients	423	Inpatient and outpatient	12%	40%	US
Miyashita et al. [22]	All cancer patients	334	Inpatient and outpatient	11%	11% (intubation only)	US
Mehta et al. [33]	All cancer patients	218	Inpatient and outpatient	28%	39%	US
Kuderer et al. [34]	All cancer patients (CC19)	928	Inpatient and outpatient	13%	26%	US, Canada, Spain
Ferrari et al. [35]	All cancer patients	198	Outpatient	17%	26%	Brazil
de Melo et al. [36]	All cancer patients	181	Inpatient	33%		Brazil
Zhang et al. [37]	All cancer patients	28	Inpatient	29%	54%	China
Yang et al. [18]	All cancer patients	205	Inpatient	20%	15% (ICU)	China
Alpert et al. [38]	All active cancer patients	421	Inpatient	31%	22% (ICU)	US
Hultcrantz et al. [39]	Multiple myeloma	100	Inpatient and outpatient	22%		US
Cook et al. [40]	Multiple myeloma patients (active)	75	Inpatient and outpatient	55%		UK
Wang et al. [41]	Multiple myeloma patients	58	Inpatient and outpatient	24%	62%	US
Mehta et al. [33]	Hematologic patients	54	Inpatient and outpatient	37%		US
Malard et al. [42]	Hematologic patients	25	Inpatient	36%		France
Marcello et al. [43]	All patients	13,442	Inpatient and outpatient	28%	46%	US
Deng et al. [44]	All patients	82,719	Inpatient and outpatient	1–8%		China
Docherty et al. [45]	All patients	20,133	Inpatient	26%		UK
Richardson et al. [23]	All patients	5700	Inpatient	21%		US
Petrilli et al. [24]	All patients	4103	Inpatient and outpatient	15%	49%	US
Guan et al. [46]	All patients	1099	Inpatient and outpatient	1–4%	6.1%	China
Goyal et al. [2]	All patients	393	Inpatient	10%	33% (intubated)	US
Zhou et al. [47]	All patients	191	Inpatient	28%		China
Onder et al. [15]	All patients	22,512	Inpatient and outpatient	7%		Italy
Wu et al. [17]	All patients	44,672	Inpatient and outpatient	2%		China
Liang et al. [16]	All patients	1590	Inpatient		39%	China
Johns Hopkins University CSSE COVID-19 map [1]	All COVID positive	175,759,427	Inpatient and outpatient	2%		US

* Note that only patients with laboratory confirmed SARS-CoV-2 infection were included in the above studies except for Wang et al., Sharafeldin et al., Fillmore et al., and our study which included a non-COVID comparison group. ** Severe events were defined as hospitalization, intubation, ICU stay, or death due to COVID-19.

## Data Availability

Data are available on request due to restrictions (i.e., HIPPA).

## Supplementary Materials

The following supporting information can be downloaded at: https://www.mdpi.com/article/10.3390/curroncol29020098/s1, Table S1: Summary of COVID-related deaths.

## References

[1] COVID-19 Map Johns Hopkins Coronavirus Resource Center. https://coronavirus.jhu.edu/map.html.

[2] Goyal P., Choi J.J., Pinheiro L.C., Schenck E.J., Chen R., Jabri A., Satlin M.J., Campion T.R., Nahid M., Ringel J.B. (2020). Clinical Characteristics of COVID-19 in New York City. N. Engl. J. Med..

[3] Stein R., Kim C. Video: ‘People Are Dying’: 72 Hours Inside a N.Y.C. Hospital Battling Coronavirus. The New York Times. https://www.nytimes.com/video/nyregion/100000007052136/coronavirus-elmhurst-hospital-queens.html.

[4] Rand D. (2020). Personal communication.

[5] Census Profile: NYC-Queens Community District 4--Elmhurst & South Corona PUMA, NY. Census Reporter. http://censusreporter.org/profiles/79500US3604107-nyc-queens-community-district-4-elmhurst-south-corona-puma-ny/.

[6] Yancy C.W. (2020). COVID-19 and African Americans. J. Am. Med. Assoc..

[7] Price-Haywood E.G., Burton J., Fort D., Seoane L. (2020). Hospitalization and Mortality among Black Patients and White Patients with COVID-19. N. Engl. J. Med..

[8] Jones J., Sullivan P.S., Sanchez T.H., Guest J.L., Hall E.W., Luisi N., Zlotorzynska M., Wilde G., Bradley H., Siegler A.J. (2020). Similarities and Differences in COVID-19 Awareness, Concern, and Symptoms by Race and Ethnicity in the United States: Cross-Sectional Survey. J. Med. Internet Res..

[9] Chen J.T., Krieger N. (2021). Revealing the Unequal Burden of COVID-19 by Income, Race/Ethnicity, and Household Crowding: US County Versus Zip Code Analyses. J. Public Health Manag. Pract..

[10] Zaorsky N.G., Yu J., McBride S.M., Dess R., Jackson W., Mahal B.A., Chen R., Choudhury A., Henry A., Syndikus I. (2020). Prostate Cancer Radiation Therapy Recommendations in Response to COVID-19. Adv. Radiat. Oncol..

[11] Al-Rashdan A., Roumeliotis M., Quirk S., Grendarova P., Phan T., Cao J., Logie N., Smith W., Barbera L. (2020). Adapting Radiation Therapy Treatments for Patients with Breast Cancer During the COVID-19 Pandemic: Hypo-Fractionation and Accelerated Partial Breast Irradiation to Address World Health Organization Recommendations. Adv. Radiat. Oncol..

[12] Spencer K., Jones C.M., Girdler R., Roe C., Sharpe M., Lawton S., Miller L., Lewis P., Evans M., Sebag-Montefiore D. (2021). The impact of the COVID-19 pandemic on radiotherapy services in England, UK: A population-based study. Lancet Oncol..

[13] Al-Shamsi H.O., Coomes E.A., Alrawi S. (2020). Screening for COVID-19 in Asymptomatic Patients with Cancer in a Hospital in the United Arab Emirates. JAMA Oncol..

[14] Willan J., King A.J., Hayes S., Collins G., Peniket A. (2020). Care of haematology patients in a COVID-19 epidemic. Br. J. Haematol..

[15] Onder G., Rezza G., Brusaferro S. (2020). Case-Fatality Rate and Characteristics of Patients Dying in Relation to COVID-19 in Italy. JAMA.

[16] Liang W., Guan W., Chen R., Wang W., Li J., Xu K., Li C., Ai Q., Lu W., Liang H. (2020). Cancer patients in SARS-CoV-2 infection: A nationwide analysis in China. Lancet Oncol..

[17] Wu Z., McGoogan J.M. (2020). Characteristics of and Important Lessons from the Coronavirus Disease 2019 (COVID-19) Outbreak in China: Summary of a Report of 72,314 Cases from the Chinese Center for Disease Control and Prevention. JAMA.

[18] Yang K., Sheng Y., Huang C., Jin Y., Xiong N., Jiang K., Lu H., Liu J., Yang J., Dong Y. (2020). Clinical characteristics, outcomes, and risk factors for mortality in patients with cancer and COVID-19 in Hubei, China: A multicentre, retrospective, cohort study. Lancet Oncol..

[19] Dai M., Liu D., Liu M., Zhou F., Li G., Chen Z., Zhang Z., You H., Wu M., Zheng Q. (2020). Patients with cancer appear more vulnerable to SARS-CoV-2: A multicenter study during the COVID-19 outbreak. Cancer Discov..

[20] Lee L.Y.W., Cazier J.-B., Angelis V., Arnold R., Bisht V., Campton N.A., Chackathayil J., Cheng V.W.T., Curley H.M., Fittall M.W.T. (2020). COVID-19 mortality in patients with cancer on chemotherapy or other anticancer treatments: A prospective cohort study. Lancet.

[21] Robilotti E.V., Babady N.E., Mead P.A., Rolling T., Perez-Johnston R., Bernardes M., Bogler Y., Caldararo M., Figueroa C.J., Glickman M.S. (2020). Determinants of COVID-19 disease severity in patients with cancer. Nat. Med..

[22] Miyashita H., Mikami T., Chopra N., Yamada T., Chernyavsky S., Rizk D., Cruz C. (2020). Do patients with cancer have a poorer prognosis of COVID-19? An experience in New York City. Ann. Oncol..

[23] Richardson S., Hirsch J.S., Narasimhan M., Crawford J.M., McGinn T., Davidson K.W., The Northwell COVID-19 Research Consortium (2020). Presenting Characteristics, Comorbidities, and Outcomes Among 5700 Patients Hospitalized with COVID-19 in the New York City Area. JAMA.

[24] Petrilli C.M., Jones S.A., Yang J., Rajagopalan H., O’Donnell L., Chernyak Y., Tobin K.A., Cerfolio R.J., Francois F., Horwitz L.I. (2020). Factors associated with hospital admission and critical illness among 5279 people with coronavirus disease 2019 in New York City: Prospective cohort study. BMJ.

[25] Chavez-MacGregor M., Lei X., Zhao H., Scheet P., Giordano S.H. (2022). Evaluation of COVID-19 Mortality and Adverse Outcomes in US Patients with or Without Cancer. JAMA Oncol..

[26] Pinato D.J., Patel M., Scotti L., Colomba E., Dolly S., Loizidou A., Chester J., Mukherjee U., Zambelli A., OnCovid Study Group (2021). Time-Dependent COVID-19 Mortality in Patients with Cancer: An Updated Analysis of the OnCovid Registry. JAMA Oncol..

[27] Wang Q., Berger N.A., Xu R. (2021). Analyses of Risk, Racial Disparity, and Outcomes among US Patients with Cancer and COVID-19 Infection. JAMA Oncol..

[28] Sharafeldin N., Bates B., Song Q., Madhira V., Yan Y., Dong S., Lee E., Kuhrt N., Shao Y.R., Liu F. (2021). Outcomes of COVID-19 in Patients with Cancer: Report from the National COVID Cohort Collaborative (N3C). J. Clin. Oncol..

[29] Fillmore N.R., La J., E Szalat R., Tuck D.P., Nguyen V., Yildirim C., Do N.V., Brophy M.T., Munshi N.C. (2021). Prevalence and Outcome of COVID-19 Infection in Cancer Patients: A National Veterans Affairs Study. J. Natl. Cancer Inst..

[30] Grivas P., Khaki A., Wise-Draper T., French B., Hennessy C., Hsu C.-Y., Shyr Y., Li X., Choueiri T., Painter C. (2021). Association of clinical factors and recent anticancer therapy with COVID-19 severity among patients with cancer: A report from the COVID-19 and Cancer Consortium. Ann. Oncol..

[31] Palmieri C., Turtle L., Docherty A., Harrison E., Drake T., Greenhalf B., Openshaw P., Baillie J., Semple M. (2020). 1670O Prospective data of first 1,797 hospitalised patients with cancer and COVID-19 derived from the COVID-19 Clinical Information Network and international Severe Acute Respiratory and emerging Infections Consortium, WHO Coronavirus Clinical Characterisation Consortium. Ann. Oncol..

[32] Rüthrich M.M., Giessen-Jung C., Borgmann S., Classen A.Y., Dolff S., Grüner B., Hanses F., Isberner N., Köhler P., on behalf of the LEOSS Study Group (2021). COVID-19 in cancer patients: Clinical characteristics and outcome—An analysis of the LEOSS registry. Ann. Hematol..

[33] Mehta V., Goel S., Kabarriti R., Cole D., Goldfinger M., Acuna-Villaorduna A., Pradhan K., Thota R., Reissman S., Sparano J.A. (2020). Case Fatality Rate of Cancer Patients with COVID-19 in a New York Hospital System. Cancer Discov..

[34] Kuderer N.M., Choueiri T.K., Shah D.P., Shyr Y., Rubinstein S.M., Rivera D.R., Shete S., Hsu C.-Y., Desai A., de Lima Lopes G. (2020). Clinical impact of COVID-19 on patients with cancer (CCC19): A cohort study. Lancet.

[35] Ferrari B.L., Gil Ferreira C., Menezes M., De Marchi P., Canedo J., de Melo A.C., Jácome A.A., Reinert T., Paes R.D., Sodré B. (2021). Determinants of COVID-19 Mortality in Patients with Cancer from a Community Oncology Practice in Brazil. JCO Glob. Oncol..

[36] De Melo A.C., Thuler L.C.S., Pecego A.C., da Silva J.L., de Albuquerque L.Z., Garrido M.M., Quintella Mendes G.L., Mendes Pereira A.C.P., Soares M.A., Viola J.P.B. (2020). Cancer inpatient with COVID-19: A report from the Brazilian National Cancer Institute. PLoS ONE.

[37] Zhang L., Zhu F., Xie L., Wang C., Wang J., Chen R., Jia P., Guan H.Q., Peng L., Chen Y. (2020). Clinical characteristics of COVID-19-infected cancer patients: A retrospective case study in three hospitals within Wuhan, China. Ann. Oncol..

[38] Alpert N., Rapp J.L., Marcellino B., Lieberman-Cribbin W., Flores R., Taioli E. (2021). Clinical Course of Cancer Patients with COVID-19: A Retrospective Cohort Study. JNCI Cancer Spectr..

[39] Hultcrantz M., Richter J., Rosenbaum C.A., Patel D., Smith E.L., Korde N., Lu S.X., Mailankody S., Shah U.A., Lesokhin A.M. (2020). COVID-19 Infections and Clinical Outcomes in Patients with Multiple Myeloma in New York City: A Cohort Study from Five Academic Centers. Blood Cancer Discov..

[40] Cook G., Ashcroft A.J., Pratt G., Popat R., Ramasamy K., Kaiser M., Jenner M., Henshaw S., Hall R., Sive J. (2020). Real-world assessment of the clinical impact of symptomatic infection with severe acute respiratory syndrome coronavirus (COVID-19 disease) in patients with multiple myeloma receiving systemic anti-cancer therapy. Br. J. Haematol..

[41] Wang B., Van Oekelen O., Mouhieddine T.H., Del Valle D.M., Richter J., Cho H.J., Richard S., Chari A., Gnjatic S., Merad M. (2020). A tertiary center experience of multiple myeloma patients with COVID-19: Lessons learned and the path forward. J. Hematol. Oncol..

[42] Malard F., Genthon A., Brissot E., Van De Wyngaert Z., Marjanovic Z., Ikhlef S., Banet A., Lapusan S., Sestilli S., Corre E. (2020). COVID-19 outcomes in patients with hematologic disease. Bone Marrow Transplant..

[43] Marcello R.K., Dolle J., Grami S., Adule R., Liu S.Y., Tatem K., Anyaogu C., Apfelroth S., Ayinla R., Boma N. (2020). Characteristics and outcomes of COVID-19 patients in New York City’s public hospital system. PLoS ONE.

[44] Deng X., Yang J., Wang W., Wang X., Zhou J., Chen Z., Li J., Chen Y., Yan H., Zhang J. (2021). Case Fatality Risk of the First Pandemic Wave of Coronavirus Disease 2019 (COVID-19) in China. Clin. Infect. Dis..

[45] Docherty A.B., Harrison E.M., Green C.A., Hardwick H.E., Pius R., Norman L., Holden K.A., Read J.M., Dondelinger F., Carson G. (2020). Features of 20 133 UK patients in hospital with COVID-19 using the ISARIC WHO Clinical Characterisation Protocol: Prospective observational cohort study. BMJ.

[46] Guan W., Ni Z., Hu Y., Liang W., Ou C., He J., Liu L., Shan H., Lei C., Hui D.S.C. (2020). Clinical Characteristics of Coronavirus Disease 2019 in China. N. Engl. J. Med..

[47] Zhou F., Yu T., Du R., Fan G., Liu Y., Liu Z., Xiang J., Wang Y., Song B., Gu X. (2020). Clinical course and risk factors for mortality of adult inpatients with COVID-19 in Wuhan, China: A retrospective cohort study. Lancet.

[48] Agyemang C., Richters A., Jolani S., Hendriks S., Zalpuri S., Yu E., Pijls B., Prins M., Stronks K., Zeegers M.P. (2021). Ethnic minority status as social determinant for COVID-19 infection, hospitalisation, severity, ICU admission and deaths in the early phase of the pandemic: A meta-analysis. BMJ Glob. Health.

[49] Workbook: NYS-COVID19-Tracker. https://covid19tracker.health.ny.gov/views/NYS-COVID19-Tracker/NYSDOHCOVID-19Tracker-Fatalities?%3Aembed=yes&%3Atoolbar=no&%3Atabs=n.

[50] Dess R.T., Hartman H.E., Mahal B.A., Soni P.D., Jackson W.C., Cooperberg M.R., Amling C.L., Aronson W.J., Kane C.J., Terris M.K. (2019). Association of Black Race with Prostate Cancer–Specific and Other-Cause Mortality. JAMA Oncol..

[51] Roetzheim R.G., Gonzalez E.C., Ferrante J.M., Pal N., Van Durme D.J., Krischer J.P. (2000). Effects of health insurance and race on breast carcinoma treatments and outcomes. Cancer.

[52] Akinyemiju T., Meng Q., Vin-Raviv N. (2016). Race/ethnicity and socio-economic differences in colorectal cancer surgery outcomes: Analysis of the nationwide inpatient sample. BMC Cancer.

[53] Park R., Lee S.A., Kim S.Y., De Melo A.C., Kasi A. (2021). Association of active oncologic treatment and risk of death in cancer patients with COVID-19: A systematic review and meta-analysis of patient data. Acta Oncol..

[54] Desai A., Gupta R., Advani S., Ouellette L., Kuderer N.M., Lyman G.H., Li A. (2021). Mortality in hospitalized patients with cancer and coronavirus disease 2019: A systematic review and meta-analysis of cohort studies. Cancer.

[55] Ramachandran P., Kathirvelu B., Chakraborti A., Gajendran M., Zhahid U., Ghanta S., Onukogu I., Narh J.T., Wang J.C., Anwer F. (2020). COVID-19 in Cancer Patients From New York City: A Comparative Single Center Retrospective Analysis. Cancer Control J. Moffitt Cancer Cent..

[56] Broom A., Kenny K., Page A., Cort N., Lipp E.S., Tan A.C., Ashley D.M., Walsh K.M., Khasraw M. (2020). The Paradoxical Effects of COVID-19 on Cancer Care: Current Context and Potential Lasting Impacts. Clin. Cancer Res..

[57] Szewczyk M., Pazdrowski J., Golusiński P., Pazdrowski P., Więckowska B., Golusiński W. (2021). The impact of the COVID-19 pandemic on the management of head and neck cancer patients at a tertiary care institution in Poland. Contemp. Oncol. Onkol..

[58] Lopez Bernal J., Andrews N., Gower C., Gallagher E., Simmons R., Thelwall S., Stowe J., Tessier E., Groves N., Dabrera G. (2021). Effectiveness of COVID-19 Vaccines against the B.1.617.2 (Delta) Variant. N. Engl. J. Med..

[59] Pajon R., Doria-Rose N.A., Shen X., Schmidt S.D., O’Dell S., McDanal C., Feng W., Tong J., Eaton A., Maglinao M. (2022). SARS-CoV-2 Omicron Variant Neutralization after mRNA-1273 Booster Vaccination. N. Engl. J. Med..

