# Supplementary material for: Healthcare Disparities and Outcomes of Cancer Patients in a Community Setting from a COVID-19 Epicenter
Source: Curr Oncol. 2022 Feb 16;29(2):1150–62. doi: 10.3390/curroncol29020098 (PMC8870882; doi:10.3390/curroncol29020098)
Supplement: Supplementary file 1 [file curroncol-29-00098-s001.zip › curroncol-1530401-supplementary.pdf]

Current Oncology

MDPI

Article

Healthcare Disparities and Outcomes of Cancer Patients in a Community Setting from a COVID-19 Epicenter

Brianna M. Jones, Eric J. Lehrer, Anurag Saraf, Zahra Shafaee, Lucas Resende Salgado and Virginia W. Osborn

Supplementary Table S1. Summary of COVID-related deaths.

Age	Sex	Race	Ethnicity	Insurance	Smoking status	BMI	Comorbidities	Tumor type	Stage	Treatment intent	Chemotherapy	Radiation	Surgery	Interval from last cancer treatment (days)
46	Male	Other	Hispanic	Medicaid	Never	20.8	Diabetes, kidney disease	Mucoepidermoid carcinoma of parotid	I	Curative	na	56 Gy/28 fx	na	13
40	Male	Other	Hispanic	Medicaid	Current	23.4	na	Squamous cell carcinoma of esophagus	III	Curative	Capecitabine Oxalplatin	50.4 Gy/28 fx	na	36
59	Male	Other	Hispanic	Medicaid	Never	32.5	Diabetes	Rectal adenocarcinoma	IIIC	Curative	Capecitabine	50.4 Gy/28 fx	na	15
77	Male	Asian	Non-Hispanic	Medicaid	Former	21.6	Diabetes, cardiovascular disease, hypertension	Squamous cell carcinoma of lung	IIIC	Curative	Paclitaxel Carboplatin	na	na	16
80	Female	Other	Hispanic	Medicare	Never	24.1	na	Myeloproliferative neoplasm	na	Curative	Hydroxyurea	na	na	1
63	Male	Other	Hispanic	Medicaid	Former	29.9	na	Adenocarcinoma of stomach	IV	Palliative	na	na	Resection	16
